# The association between weight change patterns and obesity-related complex multimorbidity: evidence from NHANES

**DOI:** 10.3389/fendo.2024.1400204

**Published:** 2024-06-21

**Authors:** Hong-Jian Gong, Xingyao Tang, Jian-Bo Zhou

**Affiliations:** ^1^ Department of Endocrinology, Beijing Tongren Hospital, Capital Medical University, Beijing, China; ^2^ Beijing Tongren Hospital, Capital Medical University, Beijing, China

**Keywords:** body weight changes, BMI, NHANES, chronic diseases, obesity

## Abstract

**Objective:**

Obesity is a major risk factor for non-communicable diseases (NCDs), which has been the leading cause of death nowadays. The aim of this study is to examine the association between total changes in body mass index (BMI) across adulthood and the risk of obesity-related complex multimorbidity in elderly, characterizing the capacity of BMI waves in predicting major chronic diseases.

**Methods:**

In this retrospective study, 15,520 participants were analyzed from the National Health and Nutrition Examination Survey (NHANES) from 1999 and 2018. BMI was categorized as obesity (≥30.0 kg/m²), overweight (25.0–29.9 kg/m²), normal weight (18.5–24.9 kg/m²), and underweight (<18.5 kg/m²). Odds ratios (ORs) with 95% confidence interval (CIs) for the relationship between BMI change patterns and major health outcomes included hypertension, cancer, chronic obstructive pulmonary disease, cardiovascular disease, and diabetes, and population attributable fractions (PAFs) of BMI were evaluated.

**Results:**

In comparison with participants who remained non-obese, those who are stable obese showed the highest risks of developing at least one chronic disease in later life, with odds ratios of 2.76 (95% CI: 2.20 to 3.45) from age 25 years to 10 years before baseline, 2.90 (2.28 to 3.68) from age 25 years to baseline, and 2.49 (2.11 to 2.95) in the 10-year period before baseline. Moving from non-obese to obese weight-change pattern in all periods (from age 25 years to 10 years before baseline: OR = 1.82; 95% CI, 1.57 to 2.11; from age 25 years to baseline: OR = 1.87; 95% CI, 1.59 to 2.19; from 10 years before baseline to baseline: OR = 1.62; 95% CI, 1.26 to 2.08) and moving from obese to non-obese, the 10-year period before baseline (OR = 1.89; 95% CI, 1.39 to 2.57) was associated with increased risk of chronic diseases. Midlife obesity status can explain the 8.6% risk of occurrence of the chronic diseases in elderly.

**Conclusions:**

Maintaining a stable healthy weight and losing weight in early adulthood and midlife are important for better life quality during the aging process. More effective strategies and policies to reduce the prevalence of obesity are needed.

## Introduction

The worldwide prevalence of obesity has nearly tripled since 1975. In 2016, over 1.9 billion adults aged 18 and above were classified as overweight, with over 650 million of them being obese (1). National strategies and policies aimed at reducing the prevalence of obesity have been far from being successful, with the number of overweight or obese children and adolescents increased more than fourfold from 4% to 18% globally (1, 2). In the absence of effective interventions, a gradual increase in the age of onset of obesity is expected, indicating that most people will gain weight during young and middle age. However, some people succeed in losing weight due to a healthy lifestyle or other reasons at the same time. As a consequence, the distinction of different weight change patterns would be important in terms of the effects of fat accumulation.

Being overweight or obese has been previously found to be associated with a marked decrease in life expectancy, surpassing tobacco as the number one lifestyle-related risk factor for premature death (3, 4). Obesity substantially increases the incidence of non-communicable diseases (NCDs) such as CVDs, hypertension, diabetes, chronic respiratory diseases [chronic obstructive pulmonary disease (COPD)], and certain types of cancer (5–7). According to the World Health Organization (WHO), 7 of the top 10 causes of death were NCDs, including ischemic heart disease, chronic respiratory diseases (COPD), diabetes, and cancer in 2019, resulting in almost three-quarters of deaths in the world and a cost of US$8.5 trillion globally each year (8, 9).

Beyond mortality and life span, the current obesity epidemic poses a challenge to healthy longevity, which calls for comprehensive assessment in the older population, comprising avoiding disease and disability, maintaining good cognitive and physical function, and remaining actively engaged in life (10). In order to better anticipate and cope with this issue, it is important to characterize the impact of energy imbalance on health problems particularly in later life. Recently, two studies, conducted separately in the US and China, investigated the impact of weight gain from early to midlife and discovered links to poorer health outcomes in later life (11, 12). In 2022, Mika et al. published the results of a 12-year follow-up study, characterizing the role of baseline obesity in the development of complex multimorbidity at older ages and finding a strong link between obesity and diverse, increasing disease burdens (13). Disappointingly, few studies have assessed the impact of changes in body mass index (BMI) over a specific time period on overall health, which better evaluates excess fat accumulation taking height into account.

Therefore, in the present study, we used data from the National Health and Nutrition Examination Survey (NHANES) database to examine the association between total changes in BMI over different periods of life and obesity-related complex multimorbidity in old age.

## Methods

Data for the study came from NHANES, a series of continuous cross-sectional surveys that provides information on the health and nutritional status of adults and children in the United States on a biannual basis. Each year, NHANES collects data from a nationally representative sample of approximately 5,000 individuals by in-person interview, mobile physical examination, and laboratory tests.

In this study, we incorporated data on adults aged 60 or above at the time of the survey from 10 cycles of consecutive NHANES 1999–2018. Baseline was defined as the time at which participants had a physical examination. Baseline weight and height were measured during physical examinations at the survey. NHANES has routinely asked questions about weight histories, including weight at age 25 and weight 10 years before the survey, which gives us a chance to capture the histories of weight change for participants. We calculated BMI using the following formula: weight (kg) divided by height (m) squared. BMIs were categorized as obesity (≥30.0 kg/m²), overweight (25.0–29.9 kg/m²), normal weight (18.5–24.9 kg/m²), and underweight (<18.5 kg/m²). Four change patterns were then generated in the primary analysis based on BMI at three time intervals (BMI_25_ to BMI_10prior_, BMI_10prior_ to BMI_baseline_, and BMI_25_ to BMI_baseline_): (1) stable non-obese pattern: BMI < 25 kg/m^2^ at both time points; (2) obese to non-obese pattern: BMI ≥ 25 kg/m^2^ at a younger age and <25 kg/m^2^ later; (3) non-obese to obese pattern: BMI < 25 kg/m^2^ at a younger age and ≥25 kg/m^2^ later; and (4) stable obese pattern: BMI ≥ 25 kg/m^2^ at both time points. This approach has been described extensively elsewhere (14). Considering the different weight statuses across each individual’s lifespan, participants were asked to recall the age at which they were at their heaviest weight. We then categorized three age_heaviest_ weight groups: under 25, 25–60, and ≥60 years.

The main outcome of the study was the obesity-related complex multimorbidity including hypertension, cancer, COPD, cardiovascular disease (CVD), and diabetes. Average blood pressure was calculated by the following protocol: The diastolic reading with zero is not used to calculate the diastolic average. If all diastolic readings were zero, then the average would be zero. If only one blood pressure reading was obtained, that reading is the average. If there is more than one blood pressure reading, the first reading is always excluded from the average. We defined cancer from a self-administered questionnaire that was completed at the clinic visit. COPD was defined as one of these following statuses: ever having received a diagnosis of “chronic bronchitis or emphysema” (15); FEV1/FVC < 0.7 post-bronchodilator; ever been told had emphysema; using drug including selective phosphodiesterase-4 inhibitors, mast cell stabilizers, leukotriene modifiers, and inhaled corticosteroids; and age above 40 with smoke history or chronic bronchitis. CVD was defined as the presence of one or more diseases of coronary heart disease, congestive heart failure, heart attack, stroke, and angina. The diagnostic criteria for diabetes are as follows: doctor-confirmed diabetes; glycohemoglobin HbA1c (%) ≥ 6.5; fasting glucose (mmol/L) ≥ 7.0; random blood glucose (mmol/L) ≥ 11.1; 2-h OGTT blood glucose (mmol/L) ≥ 11.1; and use of diabetes medication or insulin.

The following covariates associated with chronic diseases were included: age, sex, race/ethnicity, education level, annual household income, poverty, marital status, citizenship, and smoking and alcohol status. Details regarding the survey design, methods for assessment of chronic diseases, and characteristics have been described elsewhere (16).

The study did not require Institutional Review Board approval because the internal investigation was based on secondary analysis of publicly available design data (14).

### Statistical analysis

All statistical analyses were performed using R version 4.2.1 (http://www.r-project.org), and a two-tailed *p*-value of <0.05 was considered to indicate statistical significance. Demographic variables at baseline were presented as means with standard deviations or proportions.

We compared baseline characteristics according to state of chronic diseases in later life by using the *t*-test for continuous variables and *χ* ^2^ test for categorical variables. Sankey diagrams are used to depict a flow from one category of weight status to another between different time points. UpSet plot displays the intersection of multiple sets of disease combination type indicated by dots and lines. In the primary analysis, we used logistic regression models to compare the health outcomes of BMI at different time points and different weight change patterns by using the stable non-obese group as the reference (17, 18). We used logistic regression models to compare the health outcomes depending on age at heaviest weight, using the group with the heaviest weight under 25 years of age as the reference. Population attributable fractions (PAFs) measure the extent to which disease burden in some population is related to a particular behavior or exposure and is calculated by (prevalence in total population − prevalence in unexposed)/prevalence in total population (18). We used the AFglm package to calculate PAF of BMI for major health outcomes, which estimates the model-based adjusted attributable fraction for data from a logistic regression model in the form of a glm object (19). Furthermore, we repeated our analysis using 30 kg/m^2^ as the cutoff point for weight change. Model 1 was adjusted for sex and age. Model 2 was further adjusted for race/ethnicity, education level, poverty, marital status, citizenship, annual household income, and smoking and alcohol status.

## Results

**Figure 1:**
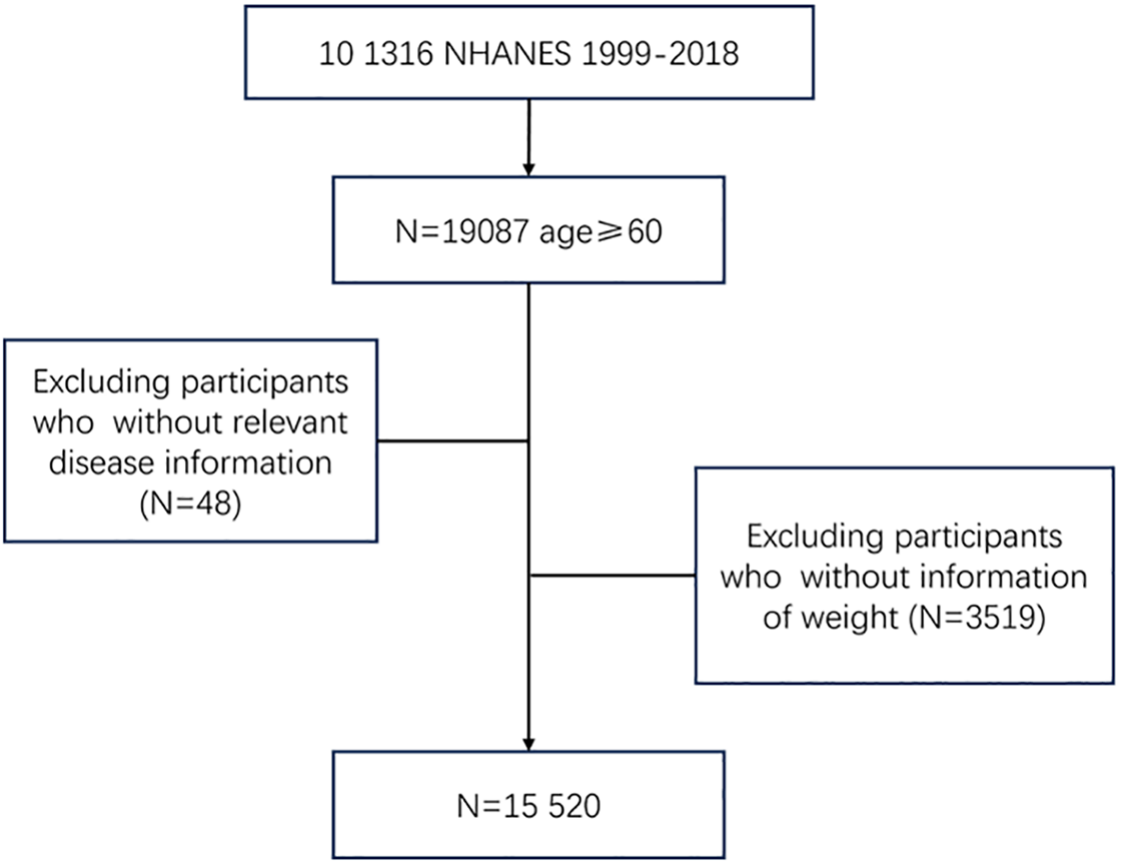
Flowchart of inclusion and exclusion of study participants.

A total of 15,520 people were included in the analysis, of whom 2,358 (15.2%) were free of COPD, CVD, cancer, hypertension, or diabetes at baseline and 13,162 (84.8%) were not. Participants were excluded if they were younger than 60 years; did not report weight; or did not have disease information (**Figure 1**). Characteristics of the study samples stratified by whether they have chronic diseases appear in **Table 1**. The mean age of the sample was 70.0 years at baseline, and 50.4% were men. The mean BMI was 22.7 kg/m^2^ at age 25, 28.2 kg/m^2^ at 10 years before the survey, and 28.9 kg/m^2^ at the baseline. Compared with people without chronic diseases, those who developed at least one of these diseases were more likely to be older, non-Hispanic White or non-Hispanic Black, poor, and less educated, and were more likely to report being in an atypical relationship and having a higher BMI throughout the life cycle; no significant differences between the two groups in terms of gender or citizenship status were found.

**Table 1:** Baseline characteristics of study participants stratified by chronic disease status. NHANES 1999–2019.^a^.

		Chronic disease at later life		
Characteristics	Total	No chronic disease	At least one chronic disease	*p*-value
Participants	15,520	2,358	13,162	–
Age, mean (SD), years	69.97 (0.10)	67.08 (0.17)	70.58 (0.10)	<0.001
Poverty, *n* (%)	3.02 (0.04)	3.34 (0.06)	2.95 (0.03)	<0.001
Age of greatest weight, mean (SD), years	56.36 (0.19)	52.92 (0.56)	57.07 (0.19)	<0.001
Number of chronic diseases, mean (SD)	1.50 (0.01)	0.00 (0.00)	1.81 (0.01)	<0.001
Mean body mass index, mean (95% CI), kg/m^2^				
At age 25 years	22.71 (0.05)	22.29 (0.09)	22.80 (0.05)	<0.001
At 10 years before baseline	28.16 (0.08)	26.29 (0.14)	28.55 (0.08)	<0.001
At survey	28.89 (0.08)	27.08 (0.14)	29.27 (0.09)	<0.001
Sex, *n* (%)				
Female	7,691 (49.56)	1,187 (56.65)	6,504 (54.34)	0.12
Male	7,829 (50.44)	1,171 (43.35)	6,658 (45.66)	
Ethnicity, *n* (%)				
Mexican American	2,017 (13)	365 (3.38)	1,652 (3.32)	<0.001
Non-Hispanic Black	3,101 (19.98)	333 (5.08)	2,768 (9.05)	
Non-Hispanic White	8,322 (53.62)	1,262 (83.41)	7,060 (79.61)	
Other Hispanic	1,095 (7.06)	206 (3.27)	889 (3.13)	
Others	985 (6.35)	192 (4.85)	793 (4.90)	
Citizenship, *n* (%)				
Citizen by birth or naturalization	14,659 (94.49)	2,168 (96.99)	12,491 (97.59)	0.07
Not a citizen of the US	854 (5.51)	188 (3.01)	666 (2.41)	
Marital status, *n* (%)				
Divorced or separated	2,279 (14.82)	375 (14.54)	1,904 (12.51)	<0.001
Married or living with partner	9,110 (59.24)	1,512 (70.02)	7,598 (62.70)	
Single	670 (4.36)	110 (3.40)	560 (3.35)	
Widowed	3,318 (21.58)	342 (12.04)	2,976 (21.44)	
Annual family income, *n* (%)				
<$65,000	11,821 (79.75)	1,670 (64.55)	10,151 (73.08)	<0.001
≥$65,000	3,002 (20.25)	571 (35.45)	2,431 (26.92)	
Education, *n* (%)				
College and above	6,900 (44.51)	1,192 (61.55)	5,708 (51.65)	<0.001
Middle and high school	6,111 (39.42)	839 (33.10)	5,272 (39.25)	
Primary school and less	2,490 (16.06)	323 (5.35)	2,167 (9.10)	

a All estimates accounted for sample weights and complex survey designs, and means and percentages were adjusted for survey weights of NHANES.

*p<0.05.

**Figure 2:**
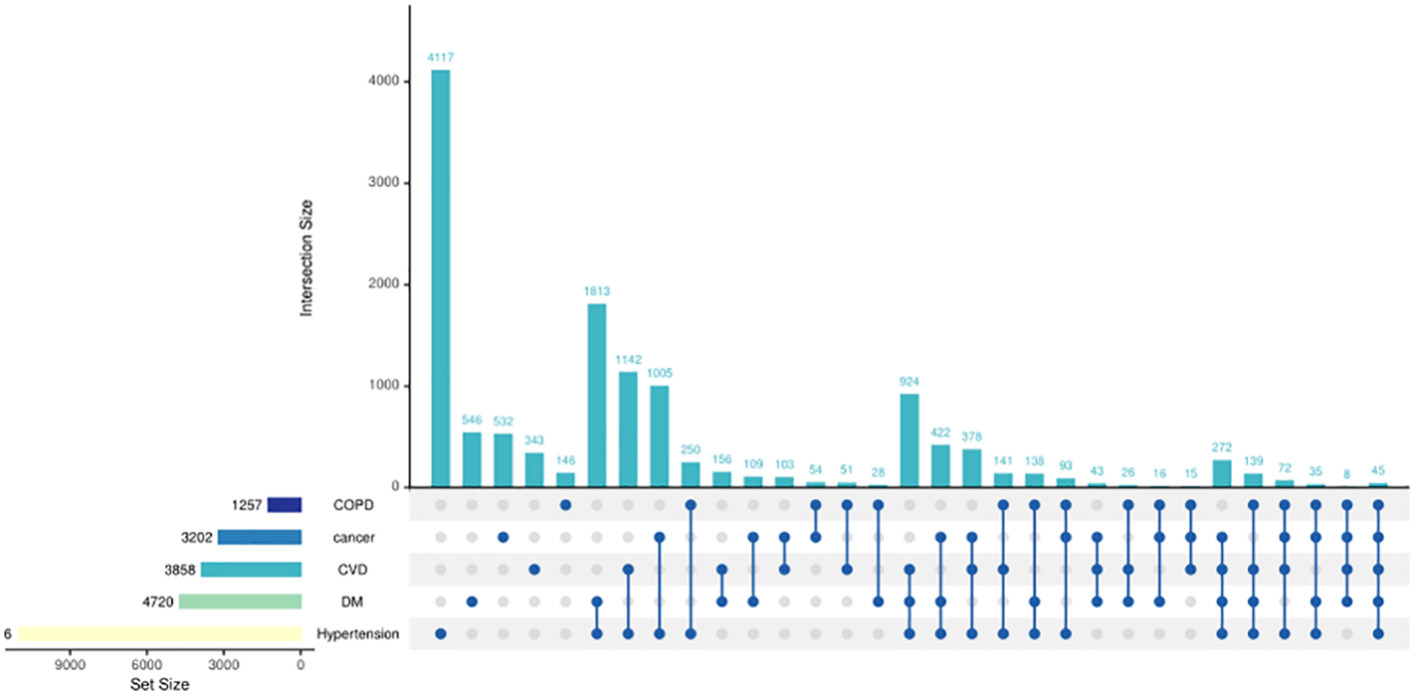
UpSet plots of chronic diseases in study participants. The presence of a single dot signifies the existence of a single disease, while the presence of two dots indicates the existence of two diseases and so on. The bar above the dots represents the number of individuals afflicted with diseases reflected by the dots. For instance, 4,117 individuals had hypertension only. The bar in the left-hand corner represents the number of individuals afflicted with a specific disease; for instance, 1,257 individuals were diagnosed with COPD.

**Figure 3:**
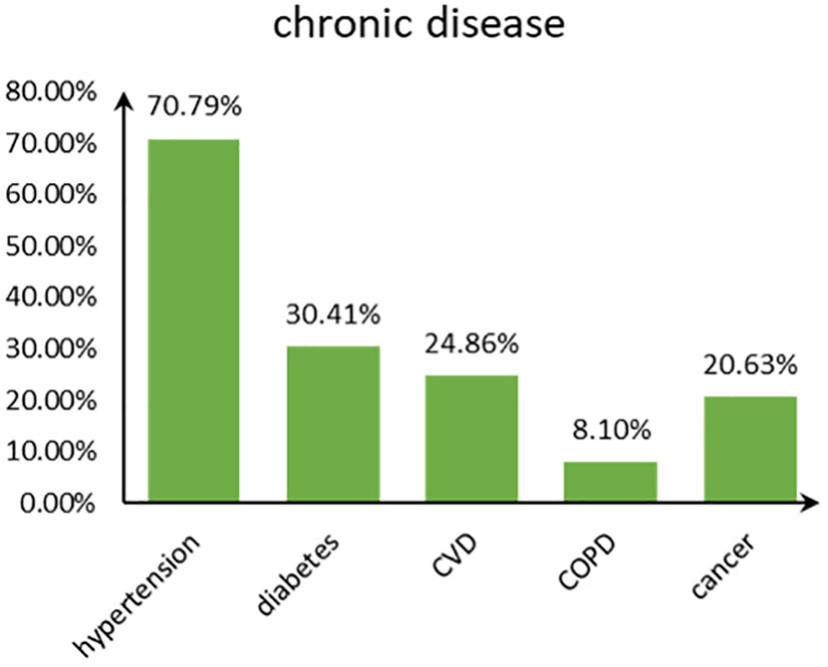
The bar chart showing the proportions of chronic diseases.

**Figure 2** shows the disease type and combinations in study participants with chronic diseases in old age. Hypertension accounted for the highest proportion among the five chronic diseases, followed by diabetes, and COPD accounted for the least (**Figure 3**). The majority suffered from hypertension only or with several comorbidities.

**Figure 4:**
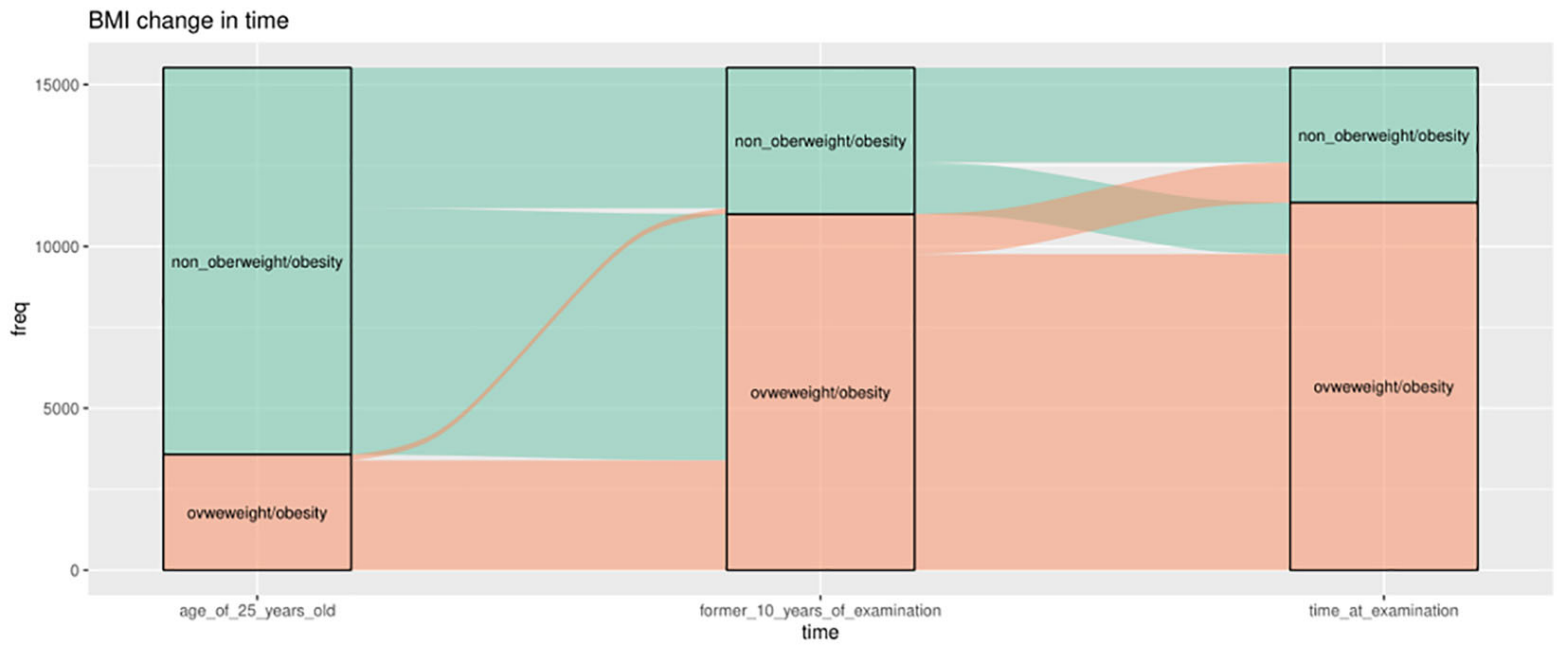
Weight change patterns across adulthood; NHANES 1999–2018 (the cutoff is 25 kg/m^2^). Three boxes represent three time points separately, 25 years old, 10 years old before baseline, and at survey time. The green color indicates those who belong to the non-overweight/obesity group (BMI ≤25 kg/m^2^), and the orange part indicates the participants who belong to the overweight/obesity group (BMI ≥25 kg/m^2^) at that time.

**Figure 4** shows the pattern of weight change using 25 kg/m^2^ as the cutoff point. At the age of 25, the majority (69.3%) had BMI levels ≤25. However, at 10 years before baseline and at examination, a significant proportion of the population (>70%) had a BMI ≥25. The majority of the population shifted from a non-obese to an obese BMI from age 25 years to 10 years before baseline, with only a small proportion shifting from the obese to the non-obese group. During the 10 years before baseline, the number of people in the obese and non-obese groups remained relatively stable, 70.9% and 73.2%, respectively. **Supplementary Figures S1**, **S2** show the weight change patterns across adulthood in NHANES 1999–2018, using two categories of weight status divided by 30 kg/m^2^ and four categories separately.

When evaluating the BMI at each time point, it was found that being overweight or obese at any time point was associated with a higher risk of major chronic diseases in later life, whereas underweight showed null association, compared with the normal group (**Table 2**). This association remained unchanged even after adjustment.

**Table 2:** Odds ratios (95% CIs) of obesity-related complex multimorbidity associated with BMI categories at different time points. NHANES 1999–2019.

	Normal	Underweight	Overweight	Obesity
25 years old				
Unadjusted	Reference	1.20 (0.93,1.55)	1.42 (1.19,1.68) ^*^	2.00 (1.29,3.11) ^*^
Model 1^a^	Reference	1.16 (0.90,1.50)	1.56 (1.29,1.89) ^*^	2.50 (1.63,3.85) ^*^
Model 2^b^	Reference	1.12 (0.84,1.49)	1.65 (1.32,2.06) ^*^	2.24 (1.41,3.56) ^*^
10 years before survey				
Unadjusted	Reference	1.20 (0.68,2.13)	1.66 (1.47,1.88) ^*^	3.07 (2.59,3.64) ^*^
Model 1^a^	Reference	1.33 (0.67,2.64)	1.62 (1.43,1.84) ^*^	3.20 (2.69,3.80) ^*^
Model 2^b^	Reference	1.38 (0.62,3.09) ^*^	1.63 (1.42,1.87) ^*^	3.09 (2.51,3.82) ^*^
At survey				
Unadjusted	Reference	1.25 (0.81,1.92)	1.39 (1.19,1.61) ^*^	2.47 (2.11,2.91) ^*^
Model 1^a^	Reference	1.17 (0.74,1.84)	1.45 (1.24,1.69) ^*^	3.03 (2.57,3.57) ^*^
Model 2^b^	Reference	1.16 (0.65,2.09) ^*^	1.48 (1.26,1.74) ^*^	3.08 (2.52,3.78) ^*^

a Model 1 was adjusted for age, sex, race/ethnicity (Mexican American, non-Hispanic black, non-Hispanic white, other Hispanic, and other race, including multi-racial), education (college and above, middle and high school, and primary school and less), and annual household income.

b Model 2 was further adjusted for drinking status (never, former, and current drinker), BMI, hypertension, smoking status (never, former, and current smoker), cardiovascular disease, diabetes, and chronic kidney disease.

^*^p<0.05.

**Table 3** shows the association between weight change patterns in the three periods and obesity-related complex multimorbidity in later life, using the stable normal group as the reference. As expected, stable obese participants were associated with the highest risks of later life chronic diseases across adulthood, with odds ratios of 2.76 (95% CI 2.20 to 3.45) from age 25 years to 10 years before baseline, 2.90 (2.28 to 3.68) from age 25 years to baseline, and 2.49 (2.11 to 2.95) in the 10-year period before baseline. Moving from the non-obese range at age 25 years to the obese range at 10 years before baseline was associated with a higher risk of chronic diseases (OR 1.82, 1.57 to 2.11), whereas the odds ratio was 1.89 (1.59 to 2.19) for the period from age 25 years to baseline and 1.62 (1.26 to 2.08) in the 10-year period before baseline. Changing from obesity to non-obesity in the 10-year period before baseline was associated with a less prevalence of chronic diseases (odds ratio 1.89, 1.39 to 2.57); the odds ratio was 0.70 (0.35 to 1.37) from age 25 years to 10 years before baseline and 1.23 (0.73 to 2.07) from age 25 years to baseline. The age of greatest weight between 25–60 and over 60 showed an association with elevated risk of chronic diseases compared with those under 25 years old, with odds ratios of 1.66 (1.28 to 2.15) and 1.93 (1.48 to 2.51).

**Table 3:** Odds ratios (95% CIs) of obesity-related complex multimorbidity associated with weight change patterns across adulthood. NHANES 1999–2018. ^c^.

Multimorbidity	Weight change patterns			
	Stable normal	Non-obese to obese	Obese to non-obese	Stable obese
From age 25 years to 10 years before baseline				
Unadjusted	Reference	1.95 (1.72,2.21) ^*^	0.70 (0.33,1.48)	2.34 (1.99,2.75) ^*^
Model 1^a^	Reference	1.89 (1.66,2.15) ^*^	0.79 (0.39,1.60)	2.65 (2.24,3.15) ^*^
Model 2^b^	Reference	1.82 (1.57,2.11) ^*^	0.70 (0.35,1.37)	2.76 (2.20,3.45) ^*^
From age 25 years to baseline				
Unadjusted	Reference	1.71 (1.48,1.96) ^*^	1.38 (0.78,2.46)	2.19 (1.82,2.64) ^*^
Model 1^a^	Reference	1.89 (1.64,2.18) ^*^	1.42 (0.81,2.49)	2.74 (2.26,3.32) ^*^
Model 2^b^	Reference	1.87 (1.59,2.19) ^*^	1.23 (0.73,2.07)	2.90 (2.28,3.68) ^*^
From 10 years before baseline to baseline				
Unadjusted	Reference	1.32 (1.05,1.67) ^*^	2.12 (1.64,2.74) ^*^	2.30 (1.99,2.66) ^*^
Model 1^a^	Reference	1.53 (1.21,1.94) ^*^	1.82 (1.41,2.35) ^*^	2.49 (2.15,2.88) ^*^
Model 2^b^	Reference	1.62 (1.26,2.08) ^*^	1.89 (1.39,2.57) ^*^	2.49 (2.11,2.95) ^*^

a Model 1 was adjusted for age, sex, race/ethnicity (Mexican American, non-Hispanic black, non-Hispanic white, other Hispanic, and other race, including multi-racial), education (college and above, middle and high school, and primary school and less), and annual household income.

b Model 2 was further adjusted for drinking status (never, former, and current drinker), BMI, hypertension, smoking status (never, former, and current smoker), cardiovascular disease, diabetes, and chronic kidney disease.

c The cutoff for non-obese and obese is 25 kg/m^2^.

^*^p<0.05.

When evaluating the association between age of the greatest BMI and major chronic diseases in later life, we identified that compared with their counterparts, participants whose age of greatest BMI between 25–60 and older than 60 was associated with a higher odds ratio of major chronic diseases, 1.66 (1.28,2.15) and 1.93 (1.48,2.51), respectively (**Table 4**).

**Table 4:** Odds ratio (95% CI) of obesity-related complex multimorbidity associated with the age of heaviest weight. NHANES 1999–2019. ^c^.

Age at heaviest weight	Under 25	Between 25 and 60	Older than 60
Unadjusted	Reference	1.39 (1.12,1.74) ^*^	2.14 (1.69,2.72) ^*^
Model 1^a^	Reference	1.75 (1.39,2.20) ^*^	2.02 (1.59,2.58) ^*^
Model 2^b^	Reference	1.66 (1.28,2.15) ^*^	1.93 (1.48,2.51) ^*^

a Model 1 was adjusted for age, sex, race/ethnicity (Mexican American, non-Hispanic black, non-Hispanic white, other Hispanic, and other race, including multi-racial), education (college and above, middle and high school, and primary school and less), and annual household income.

b Model 2 was further adjusted for drinking status (never, former, and current drinker), BMI, hypertension, smoking status (never, former, and current smoker), cardiovascular disease, diabetes, and chronic kidney disease.

c The cutoff for non-obese and obese is 25 kg/m^2^.

^*^p<0.05.

**Table 5** shows the PAFs of the BMI category at different ages for obesity-related complex multimorbidity. It is noteworthy that the 8.6% risk of occurrence of the chronic diseases can be explained by obesity in the middle-aged population, followed by the elderly (6.8%) and the young (1.3%).

**Table 5:** Population attributable fractions of overweight and obesity for obesity-related complex multimorbidity in later life by age. ^a^.

	PAF%	95% CI
BMI at 25 years old	1.3^*^	(1.0, 1.7)
BMI at 10 years before survey	8.6^*^	(7.5, 9.8)
BMI at survey	6.8^*^	(5.6, 8.0)

a The cutoff for non-obese and obese is 25 kg/m^2^.

^*^p<0.05.

In the sensitivity analysis, the major results were almost unchanged when we used 30 kg/m^2^ as the cutoff for the BMI category.

## Discussion

In the study of a large sample of the US population, we found that overweight and obesity at all ages were associated with a higher prevalence of obesity-related complex multimorbidity in later life. Crossing the threshold to overweight or obesity in all periods was linked to a higher risk of chronic disease in later life. Conversely, those who maintained a healthy BMI and those who did not experience a decline in BMI from midlife to late life had a lower risk. Weight in midlife is a stronger predictor of chronic disease, and better weight control can help reduce it by 8.6% in the population. These findings highlight the significance of maintaining a normal BMI for overall health outcomes in later life.

The Sustainable Development Goal (SDG) 3.4 makes a call to reduce premature mortality from NCDs by one-third between 2015 and 2030; NCDs severely limit people’s ability to live happy and healthy lives with their families (5). People return to poverty because of healthcare costs and labor loss, especially in low-income regions where many families have just improved their living conditions. However, as a major preventable reason of NCDs, we found that both weight gain and maintaining obesity are associated with increased risk of chronic diseases in late adulthood. Previous studies have also demonstrated the health risks associated with long-term fat accumulation (20). Increased BMI has been linked to increased risks of total mortality and mortality from various diseases (21). In a cohort study lasting 15 years, persistent obesity, developing overweight, or developing obesity were found to result in a decrease of health quality of life index for both physical and mental dimensions (22). Furthermore, weight gain during adulthood was associated with significantly increased mortality of major chronic diseases and a worsening of composite healthy aging outcomes in old age (11, 12). There are several biological mechanisms that may underlie the adverse effects of high BMI, including impaired insulin signaling and the insulin-resistant state, mechanical stress of visceral and ectopic fat storage, low-grade systemic inflammatory state, chronic overactivity of the sympathetic nervous system, and elevated bioavailable levels of insulin-like growth factor 1 and other tumor-promoting molecules (23–26). Consistent with these studies, our results reemphasize the significance of maintaining a healthy BMI in early life and midlife (21, 27, 28).

We also found that moving from the obese to the non-obese range was not associated with an increased risk of major chronic diseases compared to the normal group although they were obese at the start of the period, except for the course from midlife to late life. It indicates that individuals who are obese in early or middle age may experience improved health outcomes if they are able to achieve a weight reduction of less than 25 pounds. This finding is similar to previous studies, which reinforces the value of weight loss programs (29, 30). However, this association between weight loss and health outcomes is reversed when this occurs in the period from middle to late adulthood. The odds ratio for chronic diseases was even larger than the non-obese to obese group in this period. There are also other studies that found that becoming overweight in later life seemed to correlate with becoming healthier. A meta-analysis of 97 studies on BMI and mortality done in 2013 found no association between grade 1 obesity (30.0–34.9 kg/m^2^) and mortality, and overweight (25.0–29.9 kg/m^2^) was significantly associated with lower all-cause mortality compared to normal weight (18.5–24.9 kg/m^2^) (31). It is generally assumed that, much of the weight loss is unintentional and pathological at this stage, often a precursor to various diseases, known as the reverse causality (32–34). This is why not losing weight at this stage may mean better health. Additionally, we found that 1.3% to 8.6% of the later life chronic diseases could be attributed to being overweight/obese, with the highest attributable fraction (PAF 8.4%) in the middle age (10 years before survey). The use of the PAF to estimate the impact of being overweight or obese provides a direct public health significance, suggesting that a large proportion of people will be free from chronic diseases in later life if they can control and manage their weight. Moreover, identifying and treating overweight and obesity in midlife is of particular importance in reducing the burden of major chronic diseases. Previous researchers have found considerable PAF of excess body weight for different diseases, especially in CVDs and cancer (35–37). New evidence from the UK Biobank cohort shows that the risk of excess body weight on colorectal cancer was even higher than previously assumed (38). Our study reemphasizes and extends prior findings. However, in our study, we did not calculate PAF for each site-specific cancer because of the small number of patients with cancer, which may affect the explanation of PAF. Different cancers have varying attributable scores with BMI, and for some cancers, excess fat may show a protective effect like prostate cancer (39).

We further explore the association between age of lifetime maximum weight and major chronic diseases, finding that both between 25 and 60 years and older than 60 years are associated with increased risk of obesity-related complex multimorbidity. There are two possible explanations for this: firstly, the negative impact of obesity accumulation on chronic diseases, and secondly, premature deaths due to obesity at an early age. Regardless of the explanation, obesity is a hazardous factor, again highlighting the importance of maintaining an appropriate BMI for good health outcomes. However, the effect of early death may lead to a survival bias for PAF calculation, but because of the large population base, the overall effect of changes in weight gain should still be robust.

This study has several limitations. First, the weight at age 25 years old and 10 years before baseline was self-reported, which may have some recall errors. However, this form of recalled early life BMI, height, and weight was suggested to be a valid measure; thus, our results should remain sound (40). The second limitation is the imperfections of BMI as a metric (41), which was developed from White people. It may not quite fit people of color. BMI does not measure body fat, not to mention the distribution of fat. Third, there were very few underweight people in our study; nevertheless, a prior study found that for those with a low body weight early in life, an appropriate weight gain may be harmless or even beneficial (12). Our study did not consider this part. Furthermore, hypertension accounts for a large proportion of the outcome. Therefore, the study’s positive findings may be largely attributed to cardiovascular-related diseases. Because of the smaller portion of the population affected by COPD and cancer, and the smaller number of people with multiple comorbidities, further analysis of the association between various chronic diseases and weight, as well as other related factors, requires a larger volume of data. Finally, it is important to note that the distribution patterns of adipose tissue among the participants were not differentiated, which may vary between racial groups. Consequently, the findings of this paper are primarily applicable to European populations.

## Conclusions

To our knowledge, this is the first large study to examine the relationship between changes in BMI in lifetime and several important chronic diseases, describing estimates of different weight change patterns. Using valuable information included in weight histories, our findings demonstrate the importance of maintaining a healthy BMI as well as weight loss in early adulthood and midlife for better life quality during the aging process and call for immediate and more effective strategies and policies to reduce the prevalence of obesity. Future studies may need to combine both mortality and morbidity and use better obesity definition beyond BMI.

## Data availability statement

The original contributions presented in the study are included in the article/**Supplementary Material**. Further inquiries can be directed to the corresponding author.

## Ethics statement

Ethical review and approval was not required for the study on human participants in accordance with the local legislation and institutional requirements. Written informed consent from the patients/participants or patients/participants’ legal guardian/next of kin was not required to participate in this study in accordance with the national legislation and the institutional requirements.

## Author contributions

H-JG: Methodology, Supervision, Validation, Writing – original draft, Writing – review & editing. XT: Data curation, Formal Analysis, Methodology, Writing – original draft, Writing – review & editing. J-BZ: Conceptualization, Funding acquisition, Investigation, Supervision, Writing – review & editing.
