# Supplementary material for: The association between weight change patterns and obesity-related complex multimorbidity: evidence from NHANES
Source: Front Endocrinol (Lausanne). 2024 Jun 21;15:1400204. doi: 10.3389/fendo.2024.1400204 (PMC11224475; doi:10.3389/fendo.2024.1400204)
Supplement: Supplementary file 1 [file DataSheet_1.docx]

**Supplement Materials**

SFigure 1. Weight change patterns across adulthood in NHANES 1999-2018 (the cut-off is 30 kg/m^2^)

SFigure 2: Weight change patterns across adulthood in NHANES 1999-2018 (4 classification)

STable 1. Odds ratios (95% CIs) of obesity-related complex multimorbidity with weight change patterns across adulthood in NHANES 1999-2018 ^d^

SFigure 1. Weight change patterns across adulthood in NHANES 1999-2018 (the cut-off is 30 kg/m^2^)


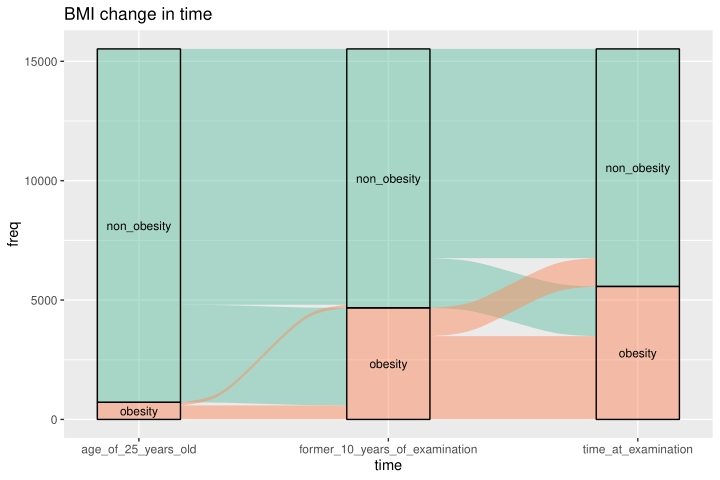


SFigure 2: Weight change patterns across adulthood in NHANES 1999-2018 (4 classification)


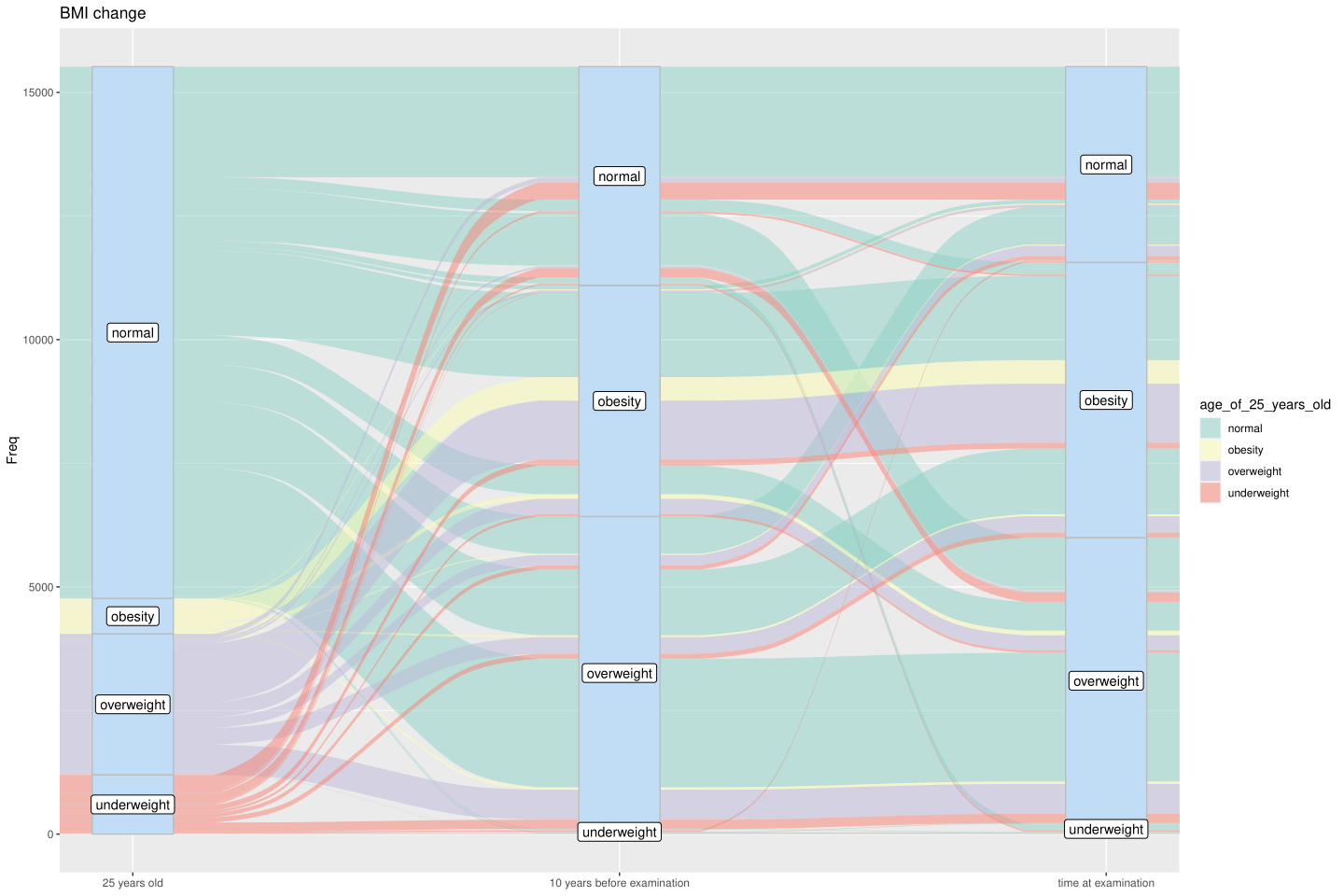


STable 1. Odds ratios (95% CIs) of obesity-related complex multimorbidity with weight change patterns across adulthood in NHANES 1999-2018 ^d^

|  | Weight change patterns | | | |
| --- | --- | --- | --- | --- |
|  | Stable normal | Non-obese to obese | Obese to non-obese | Stable obese |
| From age 25 years to 10 years before baseline | | | | |
| unadjusted | References | 2.36(1.99,2.80) ^*^ | 1.80(0.74,4.37) | 2.34(1.37,3.98) ^*^ |
| Model 1 | References | 2.41(2.02,2.86) ^*^ | 1.68(0.71,3.98) | 2.98(1.76,5.04) ^*^ |
| Model 2 | References | 2.33(1.89,2.86) ^*^ | 1.39(0.55,3.50) | 2.68(1.51,4.76) ^*^ |
| From age 25 years to baseline | | | | |
| unadjusted | References | 2.03(1.78,2.32) ^*^ | 1.71(0.90,3.26) | 2.51(1.39,4.52) ^*^ |
| Model 1 | References | 2.39(2.09,2.74) ^*^ | 1.73(0.90,3.29) | 3.45(1.94,6.16) ^*^ |
| Model 2 | References | 2.40(2.04,2.82) ^*^ | 1.39(0.67,2.87) | 3.16(1.69,5.90) ^*^ |
| From 10 years before baseline to baseline | | | | |
| unadjusted | References | 1.73(1.43,2.09) ^*^ | 2.43(1.83,3.21) ^*^ | 2.62(2.21,3.10) ^*^ |
| Model 1 | References | 2.14(1.77,2.59) ^*^ | 2.27(1.71,3.01) ^*^ | 3.01(2.53,3.58) ^*^ |
| Model 2 | References | 2.11(1.71,2.60) ^*^ | 1.95(1.44,2.65) ^*^ | 2.99(2.42,3.69) ^*^ |

^a^ Model 1 was adjusted for age, sex, race/ethnicity (Mexican American, non-Hispanic black, non-Hispanic white, other Hispanic, other race-including multi-Racial), education (college and above, middle and high school, primary school and less), annual-household-income.

^b^ Model 2 was further adjusted for drinking status (never, former, and current drinker), BMI, hypertension, smoking status (never, former, and current smoker), cardiovascular disease, diabetes, and chronic kidney disease.

^d^ the cut-off for non-obese and obese is 30 kg/m^2^

^*^ p＜0.05
